# Short-read genome sequencing at population scale: diagnostic insights from 2317 patients

**DOI:** 10.1038/s41431-026-02089-8

**Published:** 2026-03-31

**Authors:** Søren L. Faergeman, Lotte Andreasen, Naja Becher, Mette Christiansen, Claus H. Gravholt, Uffe B. Jensen, Jens Magnus Bernth Jensen, Ole H. Larsen, Sara Markholt, Katrine S. Sandgaard, Søren Vang, Dorte L. Lildballe

**Affiliations:** 1 Department of Molecular Medicine, Aarhus University Hospital, Aarhus, Denmark; 2 Department of Clinical Genetics, Aarhus University Hospital, Aarhus, Denmark; 3 Institute of Clinical Medicine; Aarhus University, Aarhus, Denmark; 4 Department of Endocrinology, Aarhus University Hospital, Aarhus, Denmark; 5 Department of Clinical Immunology, Aarhus University Hospital, Aarhus, Denmark

**Keywords:** Genetic testing, Genetics research

## Abstract

As part of the Danish National Genome Centre (DNGC) initiative, the Central Denmark Region has implemented short-read whole-genome sequencing (srWGS) as a first-tier diagnostic tool for suspected monogenetic disorders. Despite increasing adoption of genome sequencing, evidence from large-scale implementation across clinical specialties remains limited. Here, we evaluate the implementation from patient inclusion and srWGS scaling to diagnostic performance. From 2021 to 2024, we sequenced 2317 patients with suspected genetic diseases across a wide range of medical specialities. Following clinical evaluation and informed consent, Illumina srWGS was performed. Patients were categorised into clinical subgroups based on phenotype and age to support targeted variant filtration and germline variant reporting. The primary outcome was diagnostic yield across all disease groups/categories. The project is a public/private partnership co-funded by the Novo Nordisk Foundation. Diagnostic yield ranged from 6% in children with cancerto 60% in patients with skin disorders, with an overall yield of 20%. We observed substantial variation in the clinical use of srWGS as a first-tier diagnostic tool across patient categories. Regional implementation of srWGS within the DNGC framework demonstrates its scalability as a first-tier diagnostic tool for monogenic disorders. Importantly, the combination of expert-guided inclusion criteria for srWGS and mixed public/private funding has ensured equitable access to genetic diagnostics. We identify patient groups with high diagnostic returns well suited for srWGS, as well as groups where alternative strategies could also be applied.

## Introduction

Short-read Whole Genome Sequencing (srWGS) has transformed and shortened the diagnostic odyssey of patients with hereditary diseases [1]. The potential benefits are manifold: srWGS renders exome enrichment and amplification steps redundant, which reduces turnaround time in the laboratory, and importantly results in more uniform coverage of the analysed genomes. Expanding sequencing to the entire genome, improves detection of copy number variants, inversions, translocations, and repeat expansions, in addition to single nucleotide variants (SNVs) and insertions/deletions (indel) variant calling [2]. Additional genes are easily included in the diagnostic workflow and new genes can be re-analysed post initial reporting. Consequently, srWGS is becoming a first-tier clinical test that can help rule out genetic causes of disease, deliver a definitive molecular diagnosis to direct patient care, or support further analyses such as transcriptome, methylome, long-read WGS or functional analyses that may be required for variant classification [3]. However, continuous evaluation of diagnostic yields of srWGS across rare diseases is imperative to guide clinical and laboratory decision making. Specifically, delineating which patient groups benefit from genetic diagnostics based on srWGS is essential to enable comparison across diagnostic centres and for identifying those in whom causative genetic variants are likely to be missed by srWGS.

This study is based on the establishment of the Danish National Genome Centre (DNGC), a nationally coordinated, top-down initiative aimed at ensuring equitable access to srWGS-based personalised medicine in Denmark. In 2018, the Novo Nordisk Foundation granted DKK 990 million (€133 million) over a five-year period to develop and operate the DNGC’s infrastructure, to enable genome sequencing facilities in Aarhus and Copenhagen, with the aim to analyse 60,000 genomes [4]. The DNGC was officially founded in 2019 under the Danish Ministry of Health [5]. Patient group selection for srWGS involved reviewing 72 proposals and was subsequently consolidated into 18 patient categories [6]. National specialist networks of clinicians, clinical laboratory experts, representatives appointed by the Organisation of Danish Medical Societies, and patient organisations, were established to guide srWGS implementation, including defining eligible patient subgroups, providing counselling guidance, and estimating the expected number of patients suitable for srWGS [7]. A Steering Committee with representatives from key health and academic institutions oversaw the process, supported by advisory groups addressing clinical and technical aspects[7]. This structured process was intended to ensure that patient group selection was clinically relevant, feasible, and beneficial for personalised medicine[7].

The implementation of srWGS largely replaced previous methods from single gene analysis (e.g., by Sanger sequencing, MLPA or repeat-primed PCR) to targeted gene panels including exomes and array-based methods. This study presents the implementation of srWGS in the Central Denmark Region, framed by the infrastructure and legal foundation of the DNGC. The study outlines the diagnostic yield across 15 patient categories and provides a framework for scaling genomic medicine and inform the design of future diagnostic strategies.

## Materials and methods

### Sequencing infrastructure within DNGC

DNA was extracted from EDTA-stabilised peripheral blood using QIAsymphony DSP DNA Midi Kit (Qiagen, Germany) according to the manufacture’s protocol. Libraries were prepared using 300 ng input DNA with Illumina DNA PCR-Free Tagmentation protocol. A control sample was analysed for 45 common SNPs to verify sample identity. Sequencing was conducted on Illumina NovaSeq 6000 or NovaSeqX+ (Illumina, USA) (from September 2023 and onwards). The bioinformatic pipeline was created and maintained by DNGC. Briefly, reads were mapped to the hg38 reference genome [8], variants (SNVs and small indels) were called using GATK HaplotyperCaller [9], whereas use of structural variant calling developed over time and included different callers [10–13]. Repeat expansions were identified with ExpansionHunter [14], and *SMN1/SMN2* copy numbers were assessed using SMNCopyNumberCaller [15], both tools were however introduced after the study had commenced. Variants were filtered using VarSeq (Golden Helix, USA) with filtering strategies tailored to each patient group. Reported variants were classified (Classes C1–C5) according to guidelines of the American College of Medical Genetics and Genomics [16]. All laboratory work was done in ISO15189 accredited laboratories. Reported variants were only uploaded to knowledge databases such as ClinVar when patient consent was provided.

Trio analysis (mother, father, proband) was the primary strategy in five patient groups: Children and adults with rare diseases (80%), children with inborn errors of immunity (46%)foetal medicine (50%), children with cancer (95%), and in the neurogenetic patient subgroup; Children with epilepsy (95%). Others were primarily analysed as singleton analysis (proband only) (Table S1).

In patients with phenotypically well-defined rare disorders warranting a strong clinically suspicion of a genetic aetiology, we opted for a custom srWGS-based singleton approach, in which variant interpretation was narrowed down to selected genes defined by the referring clinician or the Department of Clinical Genetics.

### Patient inclusion

During the inclusion period from January 1, 2021, to September 30, 2024, a total of 2317 patients were included and sequenced by srWGS in the Central Denmark Region through the DNGC. During the study period, clinical reports were issued by one of three departments in the Central Denmark Region: Department of Clinical Immunology, Department of Clinical Genetics, or Department of Molecular Medicine. This study focus’ on all inherited diseases to allow for comparison of diagnostic yield across presumed monogenetic disorders. Somatic cancer is not included in this study. An overview of patient groups/subgroups, summary of clinical criteria and inclusion time in DNGC is provided in supplementary Table S1.

### Data collection

Information about age at testing, sex, referral reason and reported genetic findings was collected from the laboratory information systems of the reporting laboratories.

### Categorisation of reported findings

#### Explanatory findings

C4 or C5 variant(s) in a gene known to cause a phenotype matching the clinical presentation of the patient, consistent with the expected inheritance pattern (biallelic or hemizygous for recessive; monoallelic for dominant).

#### Partial findings

Heterozygous C4 or C5 variant in a gene associated with a matching recessive phenotype, but without a second variant identified.

#### Uncertain findings

C3 variants were reported based on clinical context. For patients referred for hereditary heart disease, inborn errors of immunity, hereditary cancer, haematological, or endocrinological conditions, C3 variants were routinely reported upon clinician request. For other indications, C3 variants were reported when located in a gene known to cause a phenotype matching the clinical presentation of the patient, and reclassification to either C2 or C4, based on functional or family studies, was deemed likely.

#### Secondary findings

C4 or C5 variants in genes not associated with the patient’s phenotype were reported in accordance with national guidelines from the Danish Society of Medical Genetics [17].

### Ethics

All patients provided written consent for srWGS in accordance with Danish law, which also accommodates the patient’s choice on reporting of secondary findings. Briefly, patients undergoing comprehensive genome sequencing can choose to 1) receive information on all identified secondary findings, 2) receive information only on secondary findings if there is a possibility of prevention or treatment, or 3) not to be informed about secondary findings[18]. The Scientific Ethics Committee of the Central Denmark Region evaluated the study and determined that it did not require ethics approval (case 1-10-72-103-24). The study was approved as a quality project by the hospital management at Aarhus University Hospital, Aarhus, Denmark. This approval did not permit inclusion of directly identifiable patient information, such as specific genetic variants linked to individual phenotypes.

### Statistical analysis

Statistical calculations and figure preparation for this publication were done using GraphPad Prims 10 (Dotmatics, USA).

### Large language models

ChatGPT 5.2 was used to summarise and translate clinical criteria from Danish to English in table S1.

## Results

### Implementation of srWGS

During the 45-month inclusion period, we completed srWGS and clinical reports for 2317 index patients with age and sex distributions per patient category as listed in Table 1. The initial implementation progressed slowly, with only a limited number of sequencing analyses conducted per month (see supplementary Figure 1). Extended turnaround times in the initial study period made it unfeasible to include patient groups such as foetal medicine and acutely ill children where rapid analysis is essential. As a result, the inclusion of these groups was not fully implemented until the fourth quarter of 2022 when turnaround time was reduced and considered acceptable for urgent analysis. We observed approximately equal distribution of sexes across patient categories, except in the Haematology category, which was dominated by women examined for genetic coagulopathies following excessive bleeding during labour.

**Table 1:** Overview of number of patients mean/median age and sex distribution in each patient category.

Patient group	number (%)	Age median(range)	males (%)
Hereditary heart disease	556 (24)	52 (0–86)	60
Rare diseases, children <18	520 (22)	5 (0–21)	40
Neurogenetic patients	248 (11)	42 (0–85)	49
Inborn errors of immunity	195 (8)	27 (0–71)	44
Childhood cancer	137 (6)	10 (0–29)	50
Endocrinological patients	135 (6)	33 (0–83)	45
Foetal medicine	107 (5)	NA	NA
Hereditary skin disorders	84 (4)	31 (0–74)	39
Haematology	79 (3)	41 (13–76)	20
Audiogenetics	74 (3)	5 (0–81)	50
Rare diseases, adults	74 (3)	31 (2–77)	46
Cancer in young adults and hereditary cancer in adults	45 (2)	39 (21–86)	31
Hereditary cholestatic and fibrotic liver diseases	33 (2)	35 (0–88)	48
Kidney failure	19 (1)	44 (5–61)	53
Psychiatry, children and adolescents	8 ( < 1)	11 (3–13)	56

**Fig. 1:**
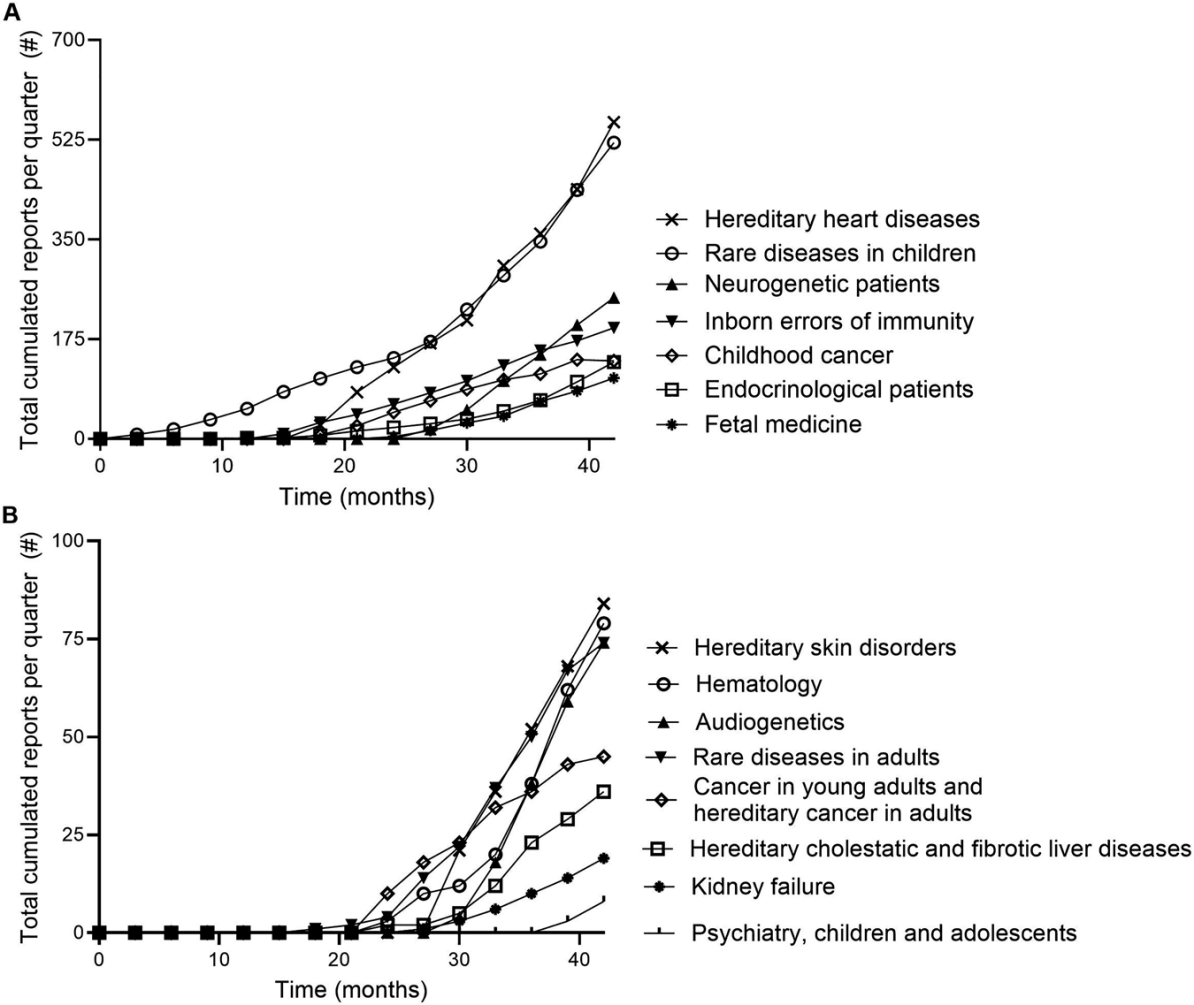
Cumulated number of patients by patient group. **A** Patient groups containing more than 100 patients in total. **B** Patient groups containing less than 100 patients in total. The patient groups were included in DNGC at different time points, see supplementary table S1. Month no. 1: January 2021.

Despite the ambition to initiate srWGS-based diagnostics simultaneously across patient categories, we observed marked differences in implementation (Fig. 1a, b). For example, hereditary heart disease and rare diseases among children <18 years of age were the most frequently represented clinical indications among the patients very early on, whereas other diseases such as psychiatric disease and kidney disease were less commonly present among sequenced patients.

During the study period, the sequencing costs were reduced significantly, from 1150 USD per sample (based on 8 samples per run on NovaSeq 6000 S2 flow cells) to 475 USD per sample (based on 40 samples per run on NovaSeq X Plus 25B flow cells). Such reductions in costs are generally expected when implementing new technologies [19], but in contrast, the cost of targeted sequencing was reduced by only around 10%, as this requires more manual handing and sequencing expenses account for a smaller proportion of the overall price (Internal data).

### Diagnostic yield

**Fig. 2:**
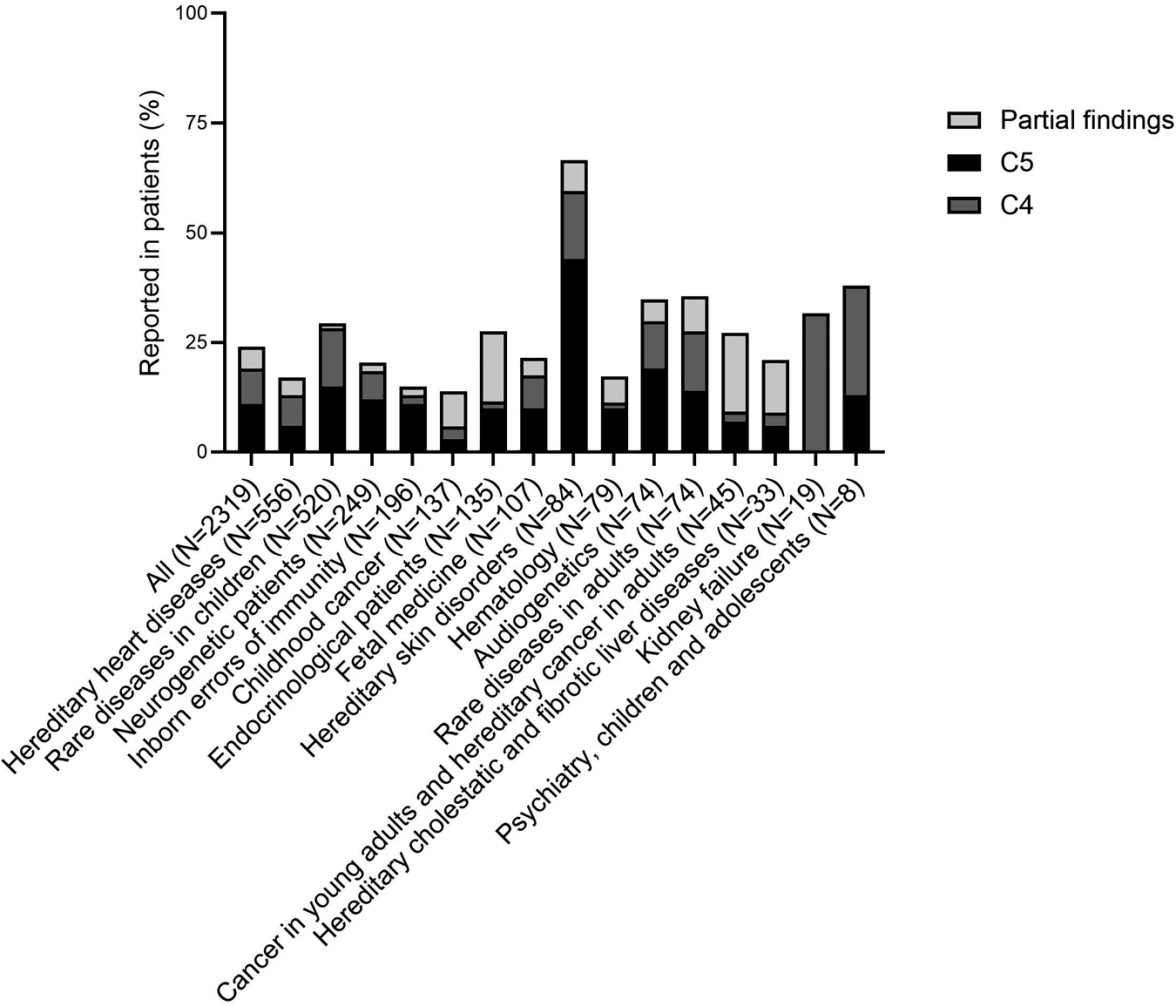
Percentage of clinical reports with explanatory findings or partial findings across patient categories. Stacked histograms displaying the proportion of test reports classified as having explanatory findings or partial findings within each patient category. *Explanatory findings* were defined as C4 or C5 variant(s) in a gene known to cause a phenotype matching that of the patient, with a genotype consistent with the expected inheritance pattern (biallelic or hemizygous for recessive; monoallelic for dominant). Partial findings refer to single heterozygous C4 or C5 variants in genes associated with a matching recessive phenotype, but without a second variant identified.

We calculated the diagnostic yield as the percentage of test reports containing explanatory findings (i.e., C4 or C5 variants consistent with both the inheritance pattern and the patient’s phenotype). The percentage of reports with partial findings, i.e., single heterozygous C4 or C5 variants in recessive genes relevant to the phenotype, were also calculated (Fig. 2). We observed an overall diagnostic yield of 20% across all patient groups, calculated as a weighted average according to the number of patients in each group, with considerable variation between patient categories. For example, the diagnostic yield of hereditary skin disorders was 60%, in contrast to only 6% in children with cancer (Fig. 2).

### Reporting of uncertain and secondary findings

**Fig. 3:**
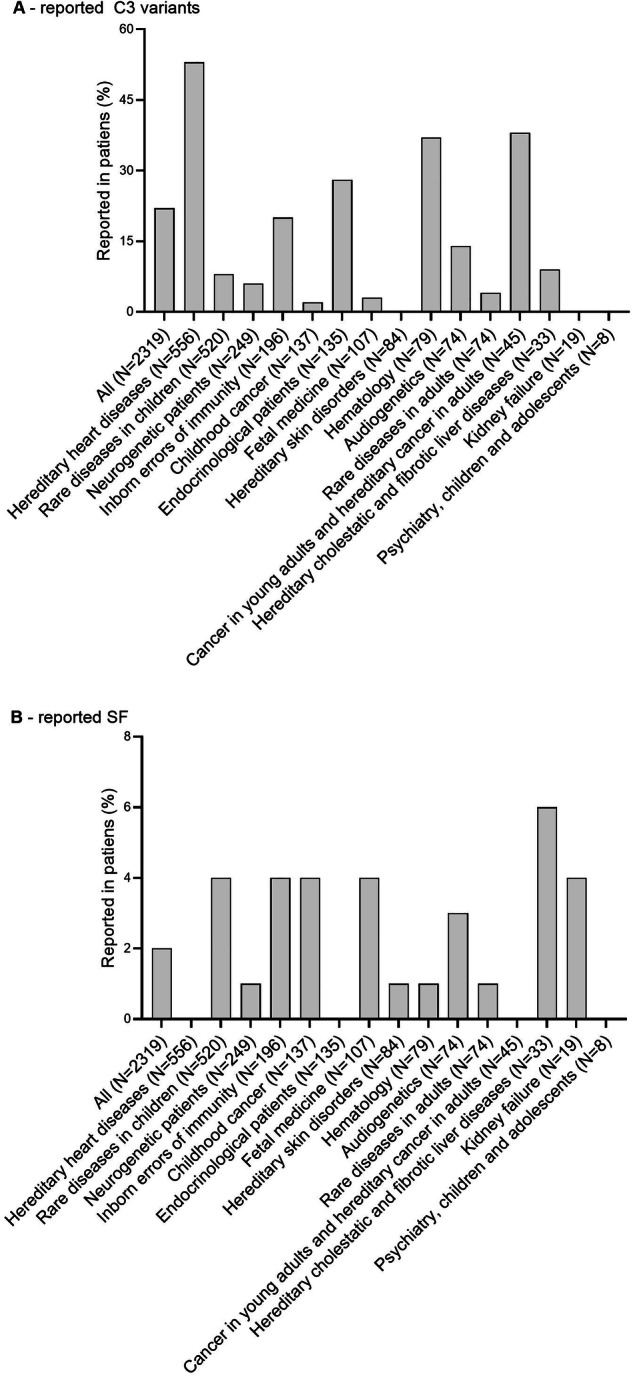
Frequency of reported uncertain and secondary findings across patient categories. Histograms depict the percentage of clinical reports with uncertain findings (i.e., reported C3 variants) (**A**) and secondary findings (i.e., C4 or C5 variants not associated with the patient’s phenotype) (**B**).

Reporting practices for uncertain findings (C3 variants) differed across patient groups, as detailed in the Methods section. Among patients with hereditary heart disease, inborn errors of immunity, hereditary cancer, haematological, or endocrine conditions, 20–50% had uncertain findings reported (Fig. 3A). In contrast, uncertain findings were reported in only 3–14% of patients in the remaining groups. Secondary findings (Fig. 3B) were reported with a low degree of variation (0–6%) across all patient categories.

### Dissecting diagnostic yields within selected patient categories

Within each patient group, the DNGC or the analysing departments defined patient subcategories with defined analytical strategies. Sequencing strategies included either trio sequencing or singleton sequencing.

**Fig. 4:**
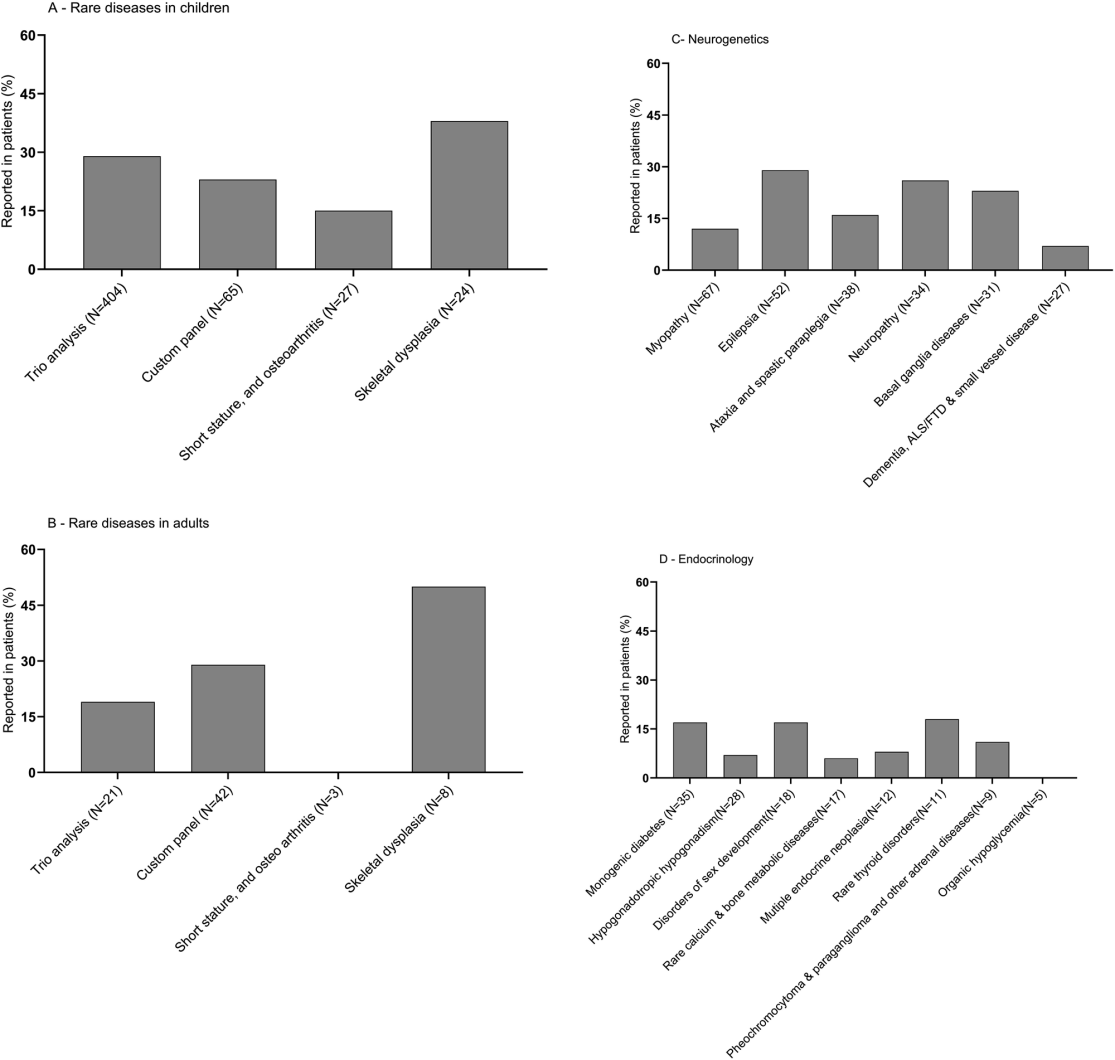
Diagnostic yield in selected patient subgroups. Diagnostic yield was defined as the number of cases with explanatory findings (C4 or C5 variant(s) in a gene relevant to the phenotype, with a genotype consistent with the expected inheritance pattern) relative to all sequenced cases within each patient category. **A** rare diseases in children, (**B**) rare diseases in adults, (**C**) neurogenetics, and (**D**) endocrinological patients.

We focused this analysis on selected patient groups (rare diseases in children and adults, neurogenetic disorders, and endocrinological patients), to evaluate variations in diagnostic yield as well as frequency of reported C3 variants and secondary findings (Fig. 4).

### Rare disease cohorts

All children suspected of having a rare disease, including malformations, neurodevelopmental or metabolic disorders in addition to monogenetic causes of acute illness, were analysed using a trio-based approach. This strategy delivered an overall diagnostic yield of 29% (Fig. 4A). Similarly, we obtained a 23% diagnostic yield using a custom gene panel singleton approach. Within specific subgroups, we observed higher diagnostic yields: 38% for children with skeletal dysplasia and 50% for adults with suspected monogenic skeletal dysplasia (though in a small cohort, *n* = 8). Diagnostic yields from both custom gene panels and trio-based analyses in adults were comparable (28% and 22%, respectively; Fig. 4B).

### Neurogenetic disorders

Explanatory findings were identified in 20% of patients overall. Subgroup analyses uncovered diagnostic yields of 23–28% in patients with epilepsy, neuropathy, or basal ganglia disease. In contrast, lower yields were found for myopathy (12%), ataxia (16%), and hereditary spastic paraplegia (19%). Among patients in the subcategory with dementia, ALS/FTD or small vessel disease, the overall yield was 7% (Fig. 4C).

### Endocrinological patients

The overall diagnostic yield was 12%. Within the eight endocrine subgroups, yields ranged from 0% in patients with organic hypoglycemia (5 patients) to almost 18% in patients disorders of sex development, monogenic diabetes (*N* = 35) and rare thyroid disorders (Fig. 4D).

## Discussion

The implementation of srWGS for a broad spectrum of diseases in Central Region Denmark has transformed and streamlined genetic testing towards srWGS. On average, the programme has delivered a genetically verified diagnosis for one in five of all analysed patients. These findings have guided clinical decision-making and facilitated genetic counselling for patients and their families. In parallel, the significant reduction in cost during the study period, has made srWGS an economically viable method in clinical genetic diagnostics. The continued decline in cost further substantiates srWGS as a first-tier test in personalised medicine.

The execution of the genomic programme has required considerable efforts by national expert panels to standardise patient inclusion criteria. Similarly, laboratory organisation, analytical workflows and pipeline design were also standardised across the country. Whilst this process reduced local flexibility, it was also a clinical support in everyday practice to have national expert panel to substantiate national consensus on clinical decisions, for example on the reporting secondary findings and C3 variants [20]. Accordingly, the standardisation has overall been successful and led to a much tighter collaboration across the country with exchange of clinical and bioinformatic information in multidisciplinary teams.

When translating this standardisation of srWGS into the clinical setting we observed differences in the adaptation to srWGS across medical specialities as reflected in Fig. 1. This was driven by several factors: If exome sequencing was used before srWGS, the barrier for changing was low, while if single gene testing was used before, the barrier was higher. Also, the customary clinical practice of requesting genetic analysis during the clinical workup of patients prior to srWGS implementation differs between clinical specialties and likely influences the number of tests requested after. Other reasons were the need to design in silico gene panels before implementing srWGS. These initial hurdles were eventually resolved through multidisciplinary team conferences with relevant clinical and laboratory stakeholders.

The new legal framework adopted into Danish law in 2019 demands written consent informing the patient about the submission of their srWGS data to the DNGC and specifying the patient’s will regarding reporting of secondary findings. This task necessitated significant clinical resources to ensure an informed consent procedure [21]. On the one hand, this was initially viewed as cumbersome and may partly have contributed to the differences in adaptation of srWGS between clinical specialties and the inclusion of patients. On the other hand, patients have become more empowered to take active part in the decision-making on which secondary findings are reported for the benefit of themselves and their families. In accordance with the Danish Society of Medical Genetics guideline, secondary findings were not actively searched for in the genomic analysis [17], which contrasts the recommendation of the American College of Medical Genetics [22]. However, we identified secondary findings in 3% of analysed index cases with limited variation across patient groups, which is comparable to similar studies [23]. Secondary findings were predominantly identified in trio analysis in which variant filtration was based on inheritance, or when analysing large gene panels, where different variants in the same genes cause different diseases. In contrast, patient groups analysed with smaller, specific gene panels (e.g. heart disease) rarely resulted in secondary findings. However, the stark differences in the reporting of secondary findings are likely also influenced by institutional practice.

The differential approach to the reporting of C3 variants was in our experience operational given that options for further analysis vary considerably between patient groups. For example, C3 variants identified in patients with Inborn errors of immunity (20%) may be further evaluated functionally, as leucocytes, the primary tissue of interest, are readily accessible for downstream testing, unlike in many other disorders. In contrast, in the prenatal setting, C3 variants are rarely reported (3%) which is supported by both national and international guidelines [24]. Nevertheless, it is important to recognise that reporting of any C3 variant risks complicating clinical decision-making and cause uncertainty among patients and their relatives [25]. In our experience, the practise of only reporting C3 when it may likely be reclassified to C2 or C4 is generally well received among patients. Future qualitative studies on patient perception and reaction to C3 reporting are warranted.

Two aspects may further reduce the number of reported C3 in the future: One is the ACMG point system that enable a distinction between “cold” and “hot” C3 variants [25] The other is the future possibilities of automated reanalysis of variants [26], which will enable that C3 variants that are reclassified to C4/C5, can be detected and clinical reports updated. Automated reanalysis may also help identifying the second pathogenic allel in a proportion of the partially solved cases.

The fraction of partially solved cases varies between the patient groups (e.g. cancer in young adults and hereditary cancer in adults in Fig. 2). This likely reflects that some gene panels included a relatively higher number of genes associated with autosomal recessive disorders, where a proportion of individuals are carriers (e.g., variants in *MUTYH*). Similarly, carrier status for hemochromatosis among endocrine patients was also frequently reported.

Delineating the diagnostic yield of each patient group has direct implications for pre-test genetic counselling, highlighting the importance of continuous monitoring of genetic testing strategies. However, patient selection and prior genetic analysis constitute likely sources of bias diagnostic yield estimates. In the first year, arrayCGH was the first-tier test in patients with rare diseases and consequently patients diagnosed by arrayCGH were not included in the study, thus introducing an underestimation of the overall diagnostic yield. Insights from comparable national genomic programmes help contextualise such diagnostic yield data. For example, the UK´s 100,000 Genomes project´s Pilot on Rare-Disease in 2183 probands, reported an overall diagnostic yield of 25% [27]. Their higher diagnostic rate compared to ours may partly be explained by the inclusion of a relatively large sub cohort with ophthalmological disorders (16% of the entire cohort), which demonstrated a markedly higher diagnostic yield (40%). Likewise, a recent study of 12,737 patients from the 2025 French Genomic Medicine Initiative generated an overall diagnostic yield of 30.6%, which was largely achieved, according to the authors, through inclusion of Malformation and Neurodevelopmental disorders, representing 62.2% of the French cohort with a diagnostic yield of 30.8%[28]. In our study, rare disease in children constituted 24% of the cohort and here we found a diagnostic yield of 28%, which is comparable to diagnostic yields in rare disease cohorts in other studies [29]. Also, the diagnostic yields of skeletal dysplasia in our cohort of paediatric and adult patients were comparable to a recent study demonstrating an overall diagnostic yield of 37–42% among children in this patient group [30]. We were surprised to find that only 19% of patients within the ataxia and spastic paraplegia patient subgroup received a genetic diagnosis. Comparable srWGS based studies have reported higher diagnostic yields 38.9% for hereditary spastic paraplegia [31] and 43% for patients with hereditary ataxia[32]. Interestingly, the authors behind this systematic review [32] note that higher diagnostic yields were associated with specific phenotype selection, suggesting a more narrow patient selection in our study might increase the diagnostic yield. In contrast, there are no studies of broadly included endocrine patients to compare with, however, we anticipate that the diagnostic yield will increase in the future with the experience gained from the current project, especially the identification of patients for genetic testing has improved during the project period. There is currently ongoing work within the European Reference Network on rare endocrine conditions to define the optimal approach to testing of endocrine patients, and a preliminary report on testing has been published [33].

As such, many factors affect the diagnostic yield of a genomic analysis, most importantly patient selection, pipeline design and variant interpretation including options for functional variant studies. Though these differentiating factors complicates direct comparison between genomic programmes, the data provides an important current baseline for the diagnostic yield of short-read sequencing-based genomic analysis.

Except for hereditary skin diseases, it is important to recognise that most of the analysed patients suspected of monogenetically driven disease remain genetically undiagnosed. Increases in the diagnostic yield may likely be achieved through better genomic data sharing infrastructure facilitated through important initiatives such as the European Genomic Data Infrastructure funded by the European Commission [34] and the Federated European Genome-Phenome Archive launched in 2022 [35]. A more stringent selection of patients with phenotypes highly suggestive of monogenetic disease will also increase the diagnostic yield albeit at the potential cost of reduced diagnostic yield in patients with less severe or atypical phenotypes.

Looking ahead, long-read sequencing is expected to provide higher diagnostic yields compared to short read sequencing primarily due to its improved ability to detect repeat expansions, variants in pseudogenes, methylation differences, and structural variants [36, 37]. It is, however, important to emphasise that also with long-read sequencing it is equally essential to harmonise national and international legal frameworks on genomic data infrastructure projects to allow for cross-border data sharing to fully harness the benefits of this technology.

## Conclusion

In conclusion, the Central Denmark Region’s implementation of srWGS demonstrates the feasibility and equity of large-scale genome sequencing as a first-tier test in routine care. Diagnostic yields across the investigated patient groups mirror comparable national genomic programmes in the UK and France, and the expert-guided harmonisation of the clinical and analytical approaches has set new standards for diagnosing monogenic disorders.

## Supplementary information

Figure S1

Table S1

Supplementary figure 1. Legend

## Data Availability

The data collected for this study is not publicly available as it contains information that could compromise patient privacy and safety. The data can, however, be made available to researchers upon application to the Danish National Committee on Health Research Ethics. Access to the data requires that the Danish National Committee on Health Research Ethics approve the requestors’ intended use of the data, and that the legal entity of the data requestor enters into a data protection agreement with the Danish data controller, the Central Denmark Region.
