# Supplementary figures and images for: Short-read genome sequencing at population scale: diagnostic insights from 2317 patients
Source: Eur J Hum Genet. 2026 Mar 31;34(6):769–76. doi: 10.1038/s41431-026-02089-8 (PMC13246910; doi:10.1038/s41431-026-02089-8)

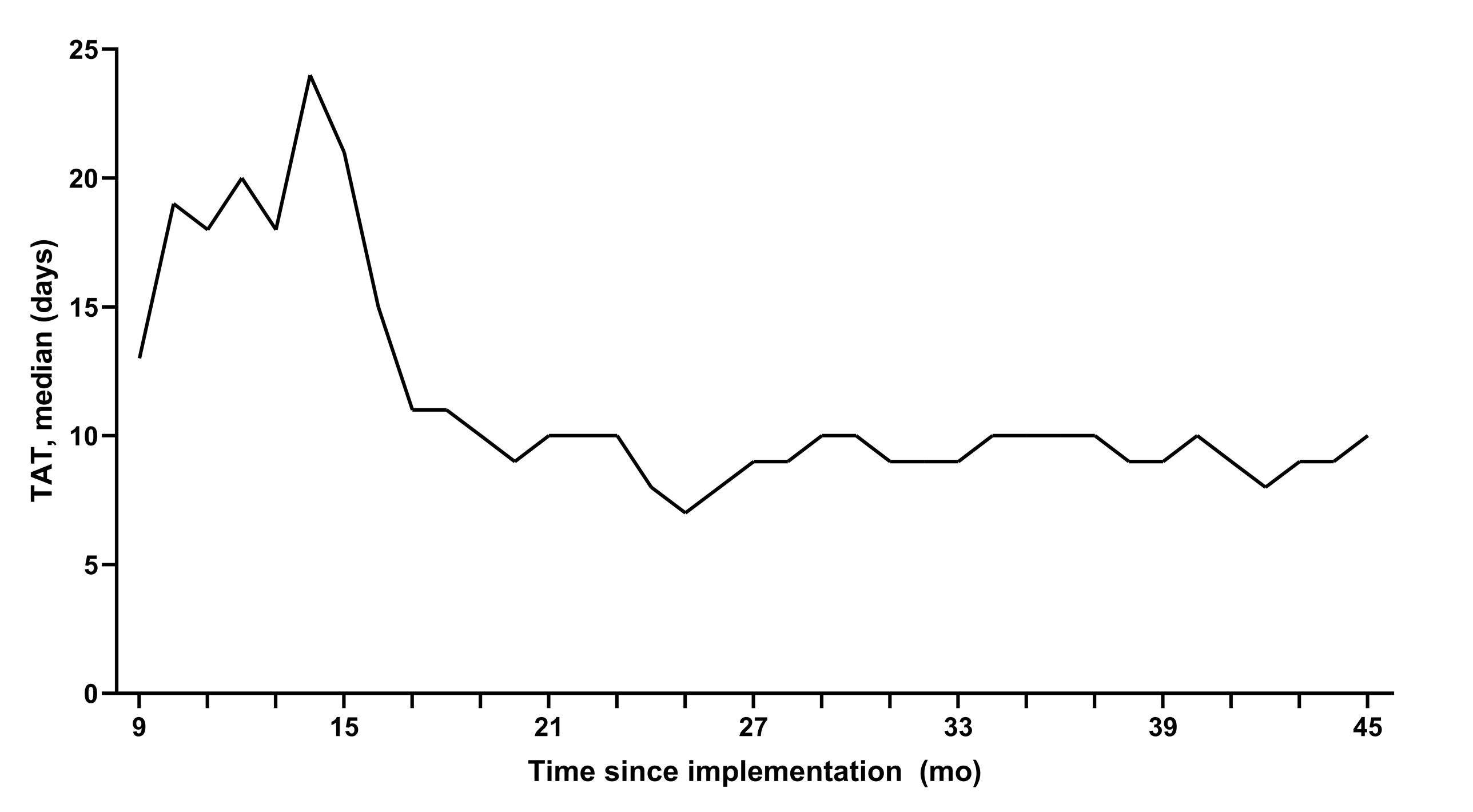

Supplement: Supplementary file 1 — Figure S1 [file 41431_2026_2089_MOESM1_ESM.jpg]
