# Supplementary material for: Short-read genome sequencing at population scale: diagnostic insights from 2317 patients
Source: Eur J Hum Genet. 2026 Mar 31;34(6):769–76. doi: 10.1038/s41431-026-02089-8 (PMC13246910; doi:10.1038/s41431-026-02089-8)
Supplement: Supplementary file 3 — Supplementary figure 1. Legend [file 41431_2026_2089_MOESM3_ESM.docx]

**Supplementary figure 1. Turnaround times in days per whole genome sequencing (WGS) equivalent from October 2021 to September 2024**. The median Turnaround times remained around 20 days until June 2022, after which it was reduced to approximately 9 days. From January to October 2021, the sample volume was too low to support weekly sequencing runs; therefore, sequencing was carried out approximately every second week to ensure economic viability.
